# Somatic cell count thresholds in composite and quarter milk samples as indicator of bovine intramammary infection status

**DOI:** 10.4102/ojvr.v84i1.1269

**Published:** 2017-03-24

**Authors:** Inge-Marié Petzer, Joanne Karzis, Edward F. Donkin, Edward C. Webb, Eric M.C. Etter

**Affiliations:** 1 Department of Production Animal Studies, University of Pretoria, South Africa; 2 Department of Animal and Wildlife Sciences, University of Pretoria, South Africa; 3 Department Environment and Societies, French Agricultural Research Centre for International Development (CIRAD), France

## Abstract

The objective of the study was to establish an operational somatic cell count (SCC) threshold to predict the presence of intramammary infection (IMI) in composite milk samples and compare findings with those in quarter milk samples. South African dairy producers now preferred composite milk samples for herd udder health analysis because of increasing cow numbers, convenience of sampling and lower cost. A retrospective study was conducted on 345 461 composite and 89 638 quarter milk samples from South African herds. Variance estimates for the proportion of quarter samples testing positive were adjusted to account for the lack of their independence within individual cows. The IMI at SCC thresholds of 150 000 cells/mL and 200 000 cells/mL differed only by 3.26% in composite milk samples. Youden’s index indicated the optimum SCC thresholds for composite and quarter milk samples as 150 000 cells/mL and 200 000 cells/mL, respectively. At 150 000 cells/mL, sensitivity (95% confidence intervals [CI]) in composite milk samples was 65.3% (64.0%, 66.6%) and specificity was 66.8% (65.7%, 67.9%); and in quarter milk samples, sensitivity at 200 000 cells/mL was 70.8% (69.5%, 72.0%) and specificity was 63.6% (62.4%, 64.8%). The likelihood of infection for udders and quarters, respectively, was 1.034 and 1.327 at an SCC threshold of 150 000 cells/mL and 0.864 cells/mL and 1.177 cells/mL at 200 000 cells/mL. The area under the curve of the receiver operating characteristics graph was 0.7084 and 0.7277 for composite and quarter samples, respectively, indicating that the SCC test could be considered as a good indicator of IMI in both sample types.

## Introduction

Bovine mastitis remains a major challenge and the disease responsible for most economic losses in dairy cows in developed countries despite improvements in management of subclinical mastitis over the past decade (Geary et al. 2012). More than 80% of financial losses because of mastitis have been estimated to occur as a result of the subclinical form of the disease indicating that this should be the key focus in proactive udder health management (Giesecke et al. 1994).

There is a correlation between the bulk milk somatic cell count (BMSCC) and the estimated percentage of cows with intramammary infection (IMI) in a herd (Smith, Hillerton & Harmon 2001). BMSCC is useful for indicating milk quality, safety and suitability for manufacturing of dairy products (Miller et al. 1993; Reneau & Packard 1991; Sheldrake, Hoare & McGregor 1983) but it does not provide information about udder health of the individual cow. No consensus exists worldwide regarding the legal limits of BMSCC in milk for human consumption. In the European Union, Australia, New Zealand, Canada and Switzerland, the legal BMSCC limit is 400 000 cells/mL; in South Africa, 500 000 cells/mL; in the USA, 750 000 cells/mL; and in Brazil, 1 million cells/mL (Ruegg & Pantoja 2013).

Somatic cell counts (SCCs) from quarter samples have been generally accepted and used as the operational measure of inflammation of the bovine lactating gland since 1960 (Barkema et al. 1999; Biggs 2009; Harmon 2001; Heeschen 1996, 2010; Schukken et al. 2003), as an indicator of the severity of IMI (Harmon 1994) and as an indicator of economic losses (DeGraves & Fetrow 1993). The SCC threshold level used to describe normal milk, IMI and subclinical mastitis, however, has often been and still is a controversial subject (Heeschen 2010). In an International Dairy Federation (IDF) bulletin (Kästli 1967), an udder quarter was considered to be normal when no pathogens were isolated and the SCC was < 500 000 cells/mL milk. However, more recently, quarter milk samples with an SCC of ≤ 100 000 cells/mL from which no microorganisms have been isolated and without a history of recent infection are considered to be normal (Harmon 2001; Smith et al. 2001), whereas those with an SCC of < 200 000 cells/mL have been regarded as an indication of an inflammatory response and that the quarter is likely to be infected (Dohoo & Leslie 1991; Laevens et al. 1997; Smith et al. 2001; Schepers et al. 1997). In 2001, the National Mastitis Council defined subclinical mastitis as an infected quarter with an SCC ≥ 200 000 cells/mL in the absence of clinical changes to milk (Smith et al. 2001) based on findings by DeGraves and Fetrow (1993), Harmon (1994) and Hillerton (1999). This decision was endorsed at an IDF World Dairy Summit in New Zealand (Harmon 2001), but it was agreed that a tolerance range of up to 400 000 cells/mL was necessary for practical reasons. Further controversy still exists in literature regarding mastitis cure, as the term ‘cure’ may be used when describing only clinical cure of mastitis, clinical cure with reduced SCC, or when clinical symptoms disappear, SCC has reduced and mastitis pathogens are absent (Hiitiö et al. 2012; Roy et al. 2012; Swinkels, Schukken & Cox 2012). The current quarter milk SCC threshold of 200 000 cells/mL does not distinguish between principal udder pathogens although pathogens are known to differ in their pathogenic effects (Barkema et al. 1999; Pantoja, Hulland & Ruegg 2009; Petzer et al. 2009, 2012). Current information on SCC threshold levels for pathogen-specific IMI is needed because SCC is known to differ between udder pathogens and over time (Petzer et al. 2009, 2012; Zadoks & Fitzpatrick 2009).

The average number of lactating cows per herd in South Africa increased from 100 in 1997 to 238 in January 2015, and in provinces, it ranges from 76 in the Northern Cape to 769 in the Eastern Cape (Lactodata 2013, 2015). Quarter milk samples for routine udder health herd examination have been replaced by composite milk samples because of practical and financial reasons. In composite samples, milk from the four udder quarters is combined, with a consequent dilution effect that has required different interpretation of the results from quarter milk samples. Although an international SCC threshold has been established for the indication of IMI in quarter milk, this has not been agreed for composite milk samples. Identification of pathogens by phenotypical classification, biochemical analysis and genotyping is costly and may well become impractical in both small and large South African herds in future. Knowledge of udder pathogens in herds remains of immense importance for a correct focus of proactive strategies and monitoring of udder health in dairy herds (Ruegg 2011; Schukken et al. 2003). Early detection of IMI will assist management, lessen the severity and duration of mastitis, reduce parenchyma damage, improve bacterial cure and lessen the risk and duration of bacterial shedding and development of new IMI (Giesecke, Du Preez & Petzer 1994; Schukken et al. 2012).

A screening test is required to enhance the likelihood of identifying individual cows at risk of having IMI within herds (Reneau & Packard 1991). Such a test can be considered successful when it is simple, inexpensive and reproducible, widely available and with sufficient discriminatory power. SCC was chosen in this study for the evaluation of composite milk samples, to be used as a screening test that could identify individual cows with possible IMI. In this article, the incidence of IMI in composite milk samples at various SCC thresholds with indications of probable accuracy (sensitivity and specificity) and an ‘optimum’ SCC threshold were evaluated. Results of composite and quarter samples were evaluated and compared to results obtained at similar SCC thresholds.

## Materials and methods

### Study design and study population

A retrospective observational study was conducted on milk samples of lactating cows from South African commercial dairy herds over a period of more than 4 years (January 2008 till April 2012). Samples originated from approximately 830 commercial South African dairy herds that submitted samples as part of their routine udder health monitoring programme and also from herds with increased BMSCC who had sought help. The lactating cow numbers in herds tested varied from approximately 30–1700 cows, whereas their intervals for herd examinations ranged from monthly and three-monthly, to annually and bi-annually, or in some cases only a once-off test.

### Data selection and sampling

In the majority of cases, all lactating cows within herds were sampled and not only selected individuals or groups. A total of 386 031 composite cow and 95 228 quarter milk samples were initially under consideration for use, of which 10.51% and 5.87%, respectively, were defined as being unsuitable and were excluded from the data set. Samples were regarded as unsuitable when any visible abnormalities such as dirt, floccules, blood or watery milk were detected. Sample results were excluded from this study when cultures indicated contaminated, mixed bacterial growth or when there were doubtful SCC results or missing identification information. The final data set comprised of 345 461 composite and 89 638 quarter milk samples. Results were exported to and captured in the Milk Sample Diagnostic (MSD) computer program and were identified by producer, cow number, quarter position, date of processing and sample type. In most cases, information regarding parity, milk yield and days in milk was not provided. The MSD program was developed over several years by Abaci South Africa for the Milk Laboratory at the University of Pretoria (Faculty of Veterinary Science) to assist in the analysis of milk samples received from dairy herds (Petzer et al. 2016). Milk samples were taken by professional samplers or milkers trained according to a standard operating procedure (Giesecke et al. 1994). Prior to sampling, first milk was stripped from all quarters and teat ends were carefully cleaned and disinfected with methylated alcohol. Approximately 10 mL of foremilk was collected aseptically into sterile marked sample tubes and kept refrigerated until shipment. In the case of composite milk samples, the same procedure was followed, only approximately equal volumes of milk from each of the four quarters were collected in one sample tube. Samples were transported on ice to reach the Milk Laboratory at the University of Pretoria (Faculty of Veterinary Science) within 48 hours after sampling. Temperatures and conditions such as sample tubes cleanliness and appearance were noted on arrival at the laboratory, and samples which were spoiled or of doubtful quality were not processed. Samples were plated out at the laboratory on the day of their arrival.

### Laboratory methods

Microbiology and SCCs were performed by the Milk Laboratory at the University of Pretoria (Faculty of Veterinary Science), on all milk samples under consideration for this study. Milk was plated on bovine blood tryptose agar plates (Oxoid, supplied by Quantum Biotechnologies [Pty] Ltd, Ferndale, South Africa). Inoculated agar plates were incubated aerobically at 37 °C ± 1 °C and examined after 18–24 hours and 48 hours. Colonies were initially identified based on colony morphology, haemolysis and potassium hydroxide test (KOH) results (IDF Document 132). The catalase reaction was used to differentiate between staphylococci and streptococci. Staphylase, a coagulase test (Oxoid, supplied by Quantum Biotechnologies [Pty] Ltd, Ferndale, South Africa), was used to distinguish between coagulase positive (*Staphylococcus aureus*) and coagulase-negative staphylococci (CNS). The Staph API test (Biomerieux South Africa [Pty] Ltd, Randburg, South Africa) was used for the identification of staphylococci. *Streptococci* species were differentiated into the various Lancefield groups using a Strepkit (Oxoid, supplied by Quantum Biotechnologies [Pty] Ltd, Ferndale, South Africa). Gram-negative organisms were diagnosed using DNase and MacConkey agar (Oxoid supplied by Quantum Biotechnologies [Pty] Ltd, Ferndale, South Africa) and API 20E (Biomerieux South Africa [Pty] Ltd, Randburg, South Africa).

IMI was defined as a pure culture when only one species grew on an inoculum. Growth of two distinct species was regarded as mixed growth, and a sample was considered to be contaminated when more than two species were present. Samples with growth of two and more colonies from the same bacterial species were recorded (Smith et al. 2001) except in the case of *S. aureus* where one and more colonies were recorded.

Somatic cells were counted by fluoro-opto-electronic means using a Fossomatic 5000 (Rhine Ruhr, P.O. Box 76167, Wendywood 2144, South Africa).

### Data analysis

In this study, all pure bacterial cultures isolated from milk samples were considered as IMIs. A further study, using the same data set investigated and analysed, results from 16 different udder pathogens. SCC was compared with the presence and absence of organisms at 11 threshold levels. Results were analysed at SCC thresholds with increments of 50 000 cells/mL up to 500 000 cells/mL, and thereafter, the increments escalated to 750 000 cells/mL and greater.

Two-by-two tables were generated. True-positive and false-positive cases were defined as those milk samples that had SCC above the level investigated and where true-positive cases were culture positive and false-positive cases were not. True-negative and false-negative samples were described as having SCC levels below the level of investigation but with either IMI (false negative) or without IMI (true negative). The results were analysed using a GenStat program (Payne et al. 2012). The likelihood ratios were determined using the category-oriented likelihood method (Moosapour et al. 2011).

In the case of composite milk samples, sensitivity, specificity and 95% confidence intervals (CI) of binomial proportions around these estimates were determined using the Mid-P exact test from the OpenEpi freeware program. Quarter samples cannot be considered to be independent but rather clustered within cows (potentially multiple sample sets per cow) (Ampe et al. 2012). Sample dependency of SCC values within each cow was modelled to take into account individual cow factors that may affect quarter samples. An estimator of cluster variance was determined as described by Scheaffer, Mendenhall and Ott (1996). The 95% confidence intervals were adjusted for clustering by using the design effect (cluster variance or sample random sample variance) (Scheaffer et al. 1996):

$$\hat{p}=\frac{\sum_{i=1}^{n}a_{i}}{\sum_{i=1}^{n}m_{i}}$$ [Eqn 1]

where $\hat{p}$ = the estimated proportion of unweighted quarters with IMI, *n* = the number of cows, a_i_ = the number of quarters with IMI per cow and *m*_i_ = the number of quarters tested per cow.

The estimated variance of the portion [$\hat{V}(\hat{p})$] of quarters with IMI was calculated using the following formula (Scheaffer et al. 1996):

$$\hat{V}(\hat{p})=\frac{\sum_{i=1}^{n}{\left(a_{i}-\hat{p}m_{i}\right)}^{2}}{n-1}$$ [Eqn 2]

The area under the curve (AUC) of the receiver operating characteristic (ROC) curves was used to estimate the discriminative power of the SCC measurement over the range of possible thresholds and was calculated with the trapezoid method (Perkins & Schisterman 2006). The best threshold point for balancing the sensitivity and specificity ratios of the SCC, the one closest to the (0.1) point, was taken as the threshold point that best differentiated between those udders (composite milk) or quarters with IMI and those without. Youden’s index which indicates the maximum distance between the ROC curve and the diagonal chance line was calculated by subtracting 1 from the sum of the maximum sensitivity and specificity.

## Results

### Composite milk samples

The SCC threshold levels of primary interest initially focused on in this study were 100 000 cells/mL, 200 000 cells/mL and 400 000 cells/mL for they were the levels in quarter milk samples that have been recommended by the International Mastitis Council (IMC) and at a Dairy World Summit. Because SCC threshold levels used on the various farms may well differ from those recommended by the IMC, other SCC ranges were also investigated in an attempt to minimise diagnostic error under different circumstances.

The percentage of IMI in this data set of 345 461 composite milk samples was 43.37% (Table 1). The percentage of samples with IMI at SCC threshold levels of 100 000 cells/mL, 200 000 cells/mL and 400 000 cells/mL was 10.89%, 18.31% and 26.26%, respectively. Of the 149 817 culture-positive samples, 57.78% had SCC in excess of 200 000 cells/ml milk compared with 26.67% of culture-negative samples. The ratio in composite samples of culture-negative to culture-positive samples was 2.960, 2.268 and 1.821 to 1 at SCC thresholds of 100 000 cells/mL, 200 000 cells/mL and 400 000 cells/mL, respectively (Table 1).

**TABLE 1:** The presence and absence of intramammary infection within each somatic cell count range in composite milk samples.

SCC range cells per mL milk	Sample numbers with IMI	Sample numbers without IMI	Total sample numbers	IMI prevalence per SCC range (95% LCI and UCI)	Cum % with IMI per SCC range
1000–50 000	19 462	77 244	96 706	0.201 (0.199, 0.204)	5, 63
51 000–100 000	18 157	34 114	52 271	0.347 (0.343, 0.352)	10, 89
101 000–150 000	14 381	19 419	33 800	0.426 (0.420, 0.431)	15, 05
151 000–200 000	11 257	12 701	23 958	0.470 (0.464, 0.476)	18, 31
201 000–250 000	9081	8055	17 136	0.530 (0.522, 0.537)	20, 94
251 000–300 000	7504	6242	13 746	0.546 (0.538, 0.554)	23, 11
301 000–350 000	6081	4445	10 526	0.578 (0.568, 0.587)	24, 87
351 000–400 000	5146	3573	8719	0.590 (0.580, 0.601)	26, 36
401 000–450 000	4280	2986	7266	0.589 (0.578, 0.600)	27, 6
451 000–500 000	3801	2520	6321	0.601 (0.589, 0.613)	28, 7
501 000–750 000	12 431	7597	20 028	0.621 (0.614, 0.627)	32, 3
750 000 +	38 236	16 748	54 984	0.695 (0.692, 0.699)	43, 37

**Totals**	**149 817**	**195 644**	**345 461**	**-**	**-**

SCC, somatic cell count; IMI, intramammary infection; LCI, lower confidence interval; UCI, upper confidence interval; Cum %, cumulative percentage.

*n* = 345 461.

Sensitivity of SCC thresholds as an indicator of IMI in composite samples was 87.0% with 95% upper and lower confidence intervals of (85.5%, 88.5%) at the lowest SCC range investigated (50 000 cells/mL) but dropped noticeably as SCC increased to 74.9% (73.5%, 76.3%) at 100 000 cells/mL, 57.8% (56.6%, 59.0%) at 200 000 cells/mL and 39.2% (38.2%, 40.2%) at 400 000 cells/mL SCC threshold ranges. At the 200 000 threshold, the specificity was 73.3% (72.2%, 74.5%) (Table 2). The likelihood ratios for culture-negative samples ranged from 3.039 at 50 000 cells/mL to 0.468 at 750 000 cells/mL, whereas the likelihood ratio became larger than 1 at the 150 000 cells/mL SCC range. The Youden’s index that balances sensitivity and specificity to obtain a mathematical optimum SCC threshold was indicated at 150 000 cells/mL in composite samples with a sensitivity of 65.3% (64.0%, 66.6%) and specificity of 66.8% (65.7%, 67.9%) (Table 2).

**TABLE 2:** Analysing intramammary infection in composite milk samples at different somatic cell count thresholds.

SCC cells per mL milk	Sensitivity (95% LCI and UCI)	Specificity (95% LCI and UCI)	Likelihood ratio	Youden’s index
50 000	0.870 (0.855, 0.885)	0.395 (0.386, 0.404)	3.039	0.265
100 000	0.749 (0.735, 0.763)	0.569 (0.559, 0.580)	1.439	0.318
150 000	0.653 (0.640, 0.666)	0.668 (0.657, 0.679)	1.034	0.321
200 000	0.578 (0.566, 0.590)	0.733 (0.722, 0.745)	0.864	0.311
250 000	0.517 (0.506, 0.529)	0.775 (0.762, 0.789)	0.679	0.292
300 000	0.467 (0.456, 0.478)	0.806 (0.794, 0.891)	0.637	0.274
350 000	0.427 (0.416, 0.437)	0.829 (0.817, 0.842)	0.560	0.256
400 000	0.392 (0.382, 0.402)	0.847 (0.837, 0.860)	0.532	0.240
450 000	0.364 (0.335, 0.373)	0.863 (0.850, 0.876)	0.534	0.226
500 000	0.338 (0.329, 0.348)	0.876 (0.863, 0.889)	0.508	0.214
750 000	0.255 (0.247, 0.263)	0.914 (0.901, 0.928)	0.468	0.170

SCC, somatic cell count; LCI, lower confidence interval; UCI, upper confidence interval.

### Quarter milk samples

Of the 89 638 quarter milk samples analysed, 43 746 (48.8%) had an SCC of above 200 000 cells/mL. The percentage of quarter milk samples in this data set from which microorganisms were isolated was 33.99% representing 30 467 milk samples (Table 3). The prevalence of IMI at various SCC ranges was determined with standard errors at 95% confidence intervals. At SCC ranges of 100 000 cells/mL, 200 000 cells/mL and 400 000 cells/mL, the prevalence (upper and lower 95% CI) of IMI was 20.4% (19.7%, 21.2%), 29.1% (28.3%, 30.9%) and 41.1% (38.9%, 43.3%).

**TABLE 3:** Quarter milk samples indicating the presence and absence of intramammary infection for each somatic cell count range.

SCC ranges cells per mL milk	Samples numbers with IMI	Samples numbers without IMI	Total sample numbers	IMI prevalence per SCC range (95% LCI and UCI)	Cum % with IMI per SCC range
1000–50 000	3019	19 891	22 910	0.132 (0.127, 0.136)	13, 18
51 000–100 000	2332	9078	11 410	0.204 (0.197, 0.212)	15, 59
101 000–150 000	1811	4923	6734	0.269 (0.258, 0.280)	17, 45
151 000–200 000	1432	3406	4838	0.291 (0.283, 0.309)	18, 73
201 000–250 000	1188	2429	3617	0.328 (0.313, 0,344)	19, 76
251 000–300 000	1068	1845	2913	0.367 (0.349, 0.384)	20, 7
301 000–350 000	899	1395	2294	0.392 (0.372, 0.412)	21, 47
351 000–400 000	790	1133	1923	0.411 (0.389, 0.433)	22, 14
401 000–450 000	711	966	1677	0.424 (0.400, 0.448)	22, 72
451 000–500 000	617	809	1426	0.433 (0.407, 0.459)	23, 21
501 000–750 000	2294	2862	5156	0.445 (0.431, 0.459)	24, 9
750 000 +	14 303	10 437	24 740	0.578 (0.572, 0.584)	33, 99

**Totals**	**30 464**	**59 174**	**89 638**	**-**	**-**

SCC, somatic cell count; IMI, intramammary infection; LCI, lower confidence interval; UCI, upper confidence interval.

*n* = 89 638.

The percentage of quarter milk samples with IMI was 18.73% at 200 000 cells/mL and 22.14% at 400 000 cells/mL. (Table 3). The ratio of quarter samples without IMI to those with IMI remained positive up to an SCC level of 750 000 cells/mL. Only at SCC above 750 000 cells/mL, there was a clear negative ratio of uninfected to infected samples.

Although discriminative measures are mostly used to make policy decisions, predictive measures are most useful in predicting the probability of a disease in an individual. The likelihood ratio for identifying culture-negative samples at various SCC thresholds ranged from 3.145 more likely at 50 000 cells/mL to 0.446 at 750 000 cells/mL. The Youden’s index at 100 000 cells/mL was 0.311 and increased to the highest values of 0.344 and 0.345 at SCC thresholds of 200 000 cells/mL and 250 000 cells/mL quarter milk (Table 4). The Youden’s index ranges from 0 for a poor to 1 for the perfect test indicating the best balance between the sensitivity and specificities of tests.

**TABLE 4:** Sensitivity and predictability of quarter intramammary infections for various somatic cell count thresholds.

SCC cells per mL milk	Sensitivity (95% LCI and UCI)^†^	Specificity (95% LCI and UCI)^†^	Likelihood ratio	Youden’s index
50 000	0.889 (0.870, 0.907)	0.336 (0.328, 0.344)	3.145	0.225
100 000	0.819 (0.808, 0.830)	0.492 (0.490, 0.503)	1.868	0.311
150 000	0.757 (0.745, 0.769)	0.577 (0.566, 0.589)	1.327	0.334
200 000	0.708 (0.695, 0.720)	0.636 (0.624, 0.648)	1.177	0.344
250 000	0.667 (0.655, 0.679)	0.678 (0.666, 0.690)	1.046	0.345
300 000	0.631 (0.618, 0.643)	0.709 (0.697, 0.722)	0.899	0.34
350 000	0.600 (0.588, 0.612)	0.734 (0.721, 0.746)	0.817	0.334
400 000	0.573 (0.561, 0.581)	0.753 (0.741, 0.765)	0.757	0.326
450 000	0.549 (0.537, 0.560)	0.770 (0.758, 0.781)	0.732	0.319
500 000	0.528 (0.516, 0.539)	0.783 (0.771, 0.795)	0.702	0.311
750 000	0.449 (0.437, 0.461)	0.882 (0.821, 0.895)	0.446	0.331

SCC, somatic cell count; LCI, lower confidence interval; UCI, upper confidence interval.

† , The effect of clustering was taken into account.

The result of the AUC of the ROC that expresses the discriminative power of the SSC test to identify IMI in both composite and quarter milk samples was 0.7084 in composite and 0.7277 in quarter milk samples (Table 5). The closer the AUC is to 1, the higher the diagnostic accuracy of the test.

**TABLE 5:** Receiver operating characteristic curves and area under the curve for the efficacy of somatic cell count test to identify intramammary infection in quarter and composite milk samples.

SCC threshold (cells per mL milk)	Q. Sen (1)	Q. Sp (0)	Q. 1-Sp (1)	Q. area	SCC threshold (cells per mL milk)	C. Sen (1)	C. Sp (0)	C. 1-Sp (1)	C. area
-	1	0.336	0.664	-	-	1	0.395	0.605	-
50 000	0.889	0.336	0.664	0.3174	50 000	0.870	0.395	0.605	0.3692
50 000	0.889	0.492	0.508	-	50 000	0.870	0.569	0.431	-
100 000	0.819	0.492	0.508	0.1332	100 000	0.749	0.569	0.431	0.1412
100 000	0.819	0.577	0.423	-	100 000	0.749	0.668	0.332	-
150 000	0.757	0.577	0.423	0.067	150 000	0.653	0.668	0.332	0.0696
150 000	0.757	0.636	0.364	-	150 000	0.653	0.733	0.267	-
200 000	0.708	0.636	0.364	0.0432	200 000	0.578	0.733	0.267	0.0399
200 000	0.708	0.678	0.322	-	200 000	0.578	0.775	0.225	-
250 000	0.667	0.678	0.322	0.0289	250 000	0.517	0.775	0.225	0.0225
250 000	0.667	0.709	0.291	-	250 000	0.517	0.806	0.194	-
300 000	0.631	0.709	0.291	0.0201	300 000	0.467	0.806	0.194	0.0157
300 000	0.631	0.734	0.266	-	300 000	0.467	0.829	0.171	-
350 000	0.600	0.734	0.266	0.0154	350 000	0.426	0.829	0.171	0.0102
350 000	0.600	0.753	0.247	-	350 000	0.426	0.847	0.153	-
400 000	0.573	0.753	0.247	0.0111	400 000	0.392	0.847	0.153	0.0075
400 000	0.573	0.770	0.230	-	400 000	0.392	0.863	0.137	-
450 000	0.549	0.770	0.230	0.0095	450 000	0.364	0.863	0.137	0.0058
450 000	0.549	0.783	0.217	-	450 000	0.364	0.876	0.124	-
500 000	0.528	0.783	0.217	0.007	500 000	0.338	0.876	0.124	0.0045
500 000	0.528	0.882	0.118	-	500 000	0.338	0.914	0.086	-
750 000	0.449	0.882	0.118	0.0484	750 000	0.255	0.914	0.086	0.0115
750 000	0.449	1	0	-	750 000	0.255	1	0	-
Maximum	0	1	0	0.0265	Maximum	0	1	0	0.0109
**AUC^†^**	-	-	-	**0.7277**	**-**	-	-	-	**0.7084**

SCC, somatic cell count; Q. Sen, quarter milk sensitivity; Q. Sp, quarter milk specificity; Q. area, quarter milk area; Q. AUC, quarter milk area under the curve; C. Sen, composite milk sensitivity; C. Sp, composite milk specificity; C. area, composite milk area; C. AUC, composite milk area under the curve.

† , Area calculated using the trapezoid method even though the graph is presented descriptively as steps (rectangles).

## Discussion

The average number of cows in South African dairy herds has increased especially in the Eastern Cape and Natal provinces (Lactodata 2015) with some herds exceeding 1700 cows. In order to be able to make effective decisions at herd and individual cow level, all lactating cows in a dairy herd were tested both for microbiology and SCC in the Milk Laboratory at the University of Pretoria (Faculty of Veterinary Science). This is, however, not the system used in other South African laboratories. Owing to the practical difficulty of sampling all cows in the large herds and analytical costs, only cows previously identified with high SCC counts or those found to have high SCC in the present test would be cultured. In some laboratories, the chosen SCC threshold for culturing may be as high as 500 000 cells/mL milk (personal communication). Information of all four quarters, all with possible different infection status, pathogen strains and inflammatory responses, is amalgamated into a composite milk sample. These results can be expected to be less informative and the values less sensitive than those from quarter milk samples. The current study has indicated that at a 500 000 cells/mL SCC threshold, 66.18% and 45.52% of IMI may be missed in composite and quarter milk samples (Tables 1 and 3).

Few test results in a laboratory are either simply positive or negative, and there is usually a gradual change between results from those considered to be normal to what may be abnormal. Dohoo and Leslie (1991) indicated that an SCC threshold of 200 000 cells/mL of milk was not an absolute value but the SCC threshold was associated with probability of IMI. Milk samples with SCC below that threshold could not be considered to be culture negative, but they were only more likely to be associated with no IMI. It was in this spirit that this large data set was analysed in order to quantify the likelihood of predicting the culture-negative samples as a whole at various SCC thresholds. This data set provided an overview of the on-farm operational conditions experienced by South African commercial dairy herds at the time. It did not attempt to distinguish between farming systems, management variations and levels, dairy breeds, cow age, parity, days in milk, quarter position, yield or stress factors originating from environmental, milking machine use, maintenance or machine settings, possible nutrition short comings, social stress or systemic diseases that may challenge the immunity of dairy cows. This study was intended to serve as an initial study to identify the relation between IMI in general and SCC levels, whereas the second part of this study will report on SCC finding regarding major and minor pathogen groups as well as for 16 specific udder pathogens.

Failure to isolate bacteria from quarter samples with high SCC does not necessarily always indicate that the udder or quarter has no IMI. Bacteria may be missed because of very low concentrations present in the milk (Ruegg & Pantoja 2013). Other studies have found that in 10% – 25% of cases of quarters with high SCC where they failed to isolate bacteria, the possibility existed that bacterial numbers were too low to detect with the laboratory technique (volume of milk used) or bacteria were excreted intermittently by the udder, as is known to occur with *S. aureus* IMI (Dohoo & Leslie 1991; Pantoja et al. 2009; Schepers et al. 1997). Schepers et al. (1997) found that 50.2% of the variation in SCC could be explained by the presence of IMI. Some other factors also known to increase SCC are days in milk, month of sampling, udder quarter position, parity, interaction between stage of lactation and parity (Schepers et al. 1997). SCC is also known to increase in the absence of IMI during stressful events such as extreme environmental temperatures. Transportation stress has been shown to change the peripheral blood neutrophil function by enhancing their migration capacity across the blood udder barrier with a consequent increase in SCC (Yagi et al. 2004). Wegner et al. (1976) reported an increase in both blood and milk leucocytes concentrations following heat stress. Green et al. (2006) in two consecutive summers found increased proportions of cows with SCC above 200 000 cells/mL without evidence of higher IMI and considered that heat stress was presumably responsible for 70.8% of the overall SCC increase. In such situations, it may not be beneficial to treat cows with intramammary antimicrobials.

### Quarter milk samples

The most accurate relationship between IMI and SCC exists where quarter milk samples are taken, and reduced milk yields have been observed and when SCC has exceeded 100 000 cells/mL (Schukken et al. 2003). SCCs of milk from healthy quarters have been found to be consistently low, and a threshold of below 200 000 cells/mL has been proposed to be the most practical level for defining udder health (Dohoo & Leslie 1991; Pantoja et al. 2009; Schepers et al. 1997).

In this study, the percentage of quarter milk samples with IMI at 100 000 (15.59%) and at 200 000 cells/mL (18.73%) differed only slightly indicating a reduced risk of missing IMI when using a threshold of 200 000 cells/mL, compared to a threshold of 100 000 cells/mL. Furthermore, over 63.03% of all quarter samples tested were culture negative at the 200 000 cells/mL SCC threshold, indicating the usefulness of SCC as a screening test in quarter milk samples. Compared with similar studies by Eberhart et al. (1979) and Malinowski et al. (2006), the percentage of uninfected quarters in this study at threshold levels of 100 000 and 200 000 cells/mL was noticeably higher than their findings of 50.0% and 59.6%, respectively.

Although the sensitivity ratio predicting accuracy in identifying IMI at 50 000 cells/mL was relatively high at 88.9% with 95% upper and lower confidence intervals of (87.0%, 90.7%), it reduced rapidly to 81.9% (80.8%, 83.0%), 70.8% (69.5%, 72.0%) and 57.3% (56.1%, 58.1%) at SCC levels of 100 000 cells/mL, 200 000 cells/mL and 400 000 cells/mL, respectively. These sensitivity ratios, although slightly lower, compared favourably with those found by Schepers et al. (1997) of 83.2%, 74.5% and 60.8% at similar SCC thresholds. The specificity that predicted accuracy of culture-negative results increased from a low of 49.2% (49.0%, 50.3%) at 100 000 cells/mL to 75.3% (74.1%, 76.5%) at 400 000 cells/mL (Table 4). Specificity values reported by this study for SCC ranges of 100 000 cells/mL, 200 000 cells/mL and 400 000 cells/mL were, however, markedly lower than the 80.5%, 89.6% and 95.0% reported by Schepers et al. (1997). Hillerton (2000) proposed sensitivity of 80% and specificity of 99% as appropriate target values for screening tests, but these were clearly impractical in this study, even for quarter milk samples. The 95% upper and lower CI for sensitivity and specificity that was adapted for clustering varied little for all SCC threshold levels, indicating that cow factors had little or no influence on the SCC levels of the quarters in this study, but it should be taken into consideration that this could possibly have been masked by the large data set.

Clinicians are often more interested in the predictive values of a test indicating a disease than in the sensitivity or specificity ratios of the test. Ruegg and Pantoja (2013) found positive predictive values relatively poor for indicating recovery from IMI when using the 200 000 SCC thresholds. The likelihood ratio (Table 4) obtained in this study confirmed that finding of Ruegg and Pantoja (2013). The likelihood ratio 1.177 at an SCC level of 200 000 cells/mL increased to below 1 only at an SCC threshold of 300 000 cells/mL. A likelihood ratio of < 1 indicates that the SCC level is more commonly associated with lack of IMI than with IMI. Although the highest Youden’s index (0.345) was found to be at an SCC threshold of 250 000 cells/mL in quarter milk, the threshold of 200 000 cells/mL with an almost similar value (0.344) was selected. This was done because of its higher sensitivity (70.8% vs. 66.7%) at an SCC of 200 000 cells/mL with the aim of identifying cases with IMI rather than those without (Table 4). Even the highest values obtained with the Youden’s index were closer to 0 than 1 indicating only fair accuracy. The efficacy of the SCC test for indicating IMI in quarter milk was found to be good but not excellent when the AUC in the ROC curve was determined at 0.7277 (Table 5).

Different populations, for example, younger cows or herds with a low or high BMSCC, may justify the use of different SCC thresholds for screening purposes. When the prevalence of IMI in a herd is high, the SCC threshold used may shift towards higher specificity levels, in order to identify the true-negative cows, whereas sensitivity might be of greater value in identifying true-positive cases in herds where IMI prevalence is low. Thus prior to analysing the data for practical operations, clarity should be obtained as to the requirement for that specific dairy herd. The operational threshold selected should depend on what the practitioner wants to achieve with this information and what is therefore the most important aspect to optimise. One of the first questions to answer is whether it is more important to identify samples with IMI or samples without IMI. Because sensitivity and specificity ratios are inversely related in most cases, one of the two will be favoured when setting a threshold in a test. When the aim is to eradicate IMI with *Streptococcus agalactiae* from a herd through separation and treatment of cows with infected udders, for instance, it could be beneficial to test all cows in the herd, even if the prevalence is low, or to set a low threshold because of the low sensitivity at higher SCC levels. When culling is the purpose for testing, as may be the case with *S. aureus*, the SCC threshold may be increased to high levels such as 800 000 cells/mL (Sol et al. 1997; Swinkels et al. 2012). In the latter case, the SCC test should rather be used as a screening and not a diagnostic test to select cows for further bacterial analysis. When a decision needs to be made whether subclinical IMI with *S. aureus* should be treated with the SCC level, the number and position of quarters infected, parity, days in milk and treatment regime known, the probable outcome of treatment success can be calculated (Sol et al. 1997). Knowledge of cows or quarters infected with CNS may be of little to no benefit and the cost of further testing may not be justified. Cows with CNS are neither treated nor separated when infected. It may therefore be beneficial to use variable SCC thresholds when evaluating dairy herds depending on the main udder pathogens isolated from that herd.

### Composite milk samples

Few studies have been conducted on composite milk samples compared with the number of studies conducted investigating SCC and IMI in quarter milk samples. A composite sample is a combination of milk from the four udder quarters, and it is to be expected that the sensitivity and specificity of this sample type should differ at similar SCC levels from that in quarter milk samples.

In this study, composite milk samples of 52.91% and 59.84% had SCC ≤ 150 000 cells/mL and ≤ 200 000 cells/mL, respectively, and 15.05% and 18.31% were culture positive at the same SCC levels (Table 1). This is a disturbingly high percentage if only viewed from a point of possible misdiagnosis of IMI when choosing either of these SCC thresholds. However, it needs to be considered that a substantial portion of these IMI may be minor pathogens and CNS. The proportion of major pathogens and contagious species isolated from low SCC was not examined in this article, but will be reported in a subsequent article.

Sensitivities of the SCC test for the composite milk samples were 65.3% with 95% CI of (64.0%, 66.6%) at 150 000 and 57.8% (56.6%, 59.0%) at 200 000 cells/mL and decreased to 25.5% (24.7%, 26.3%) at the highest SCC range. These results indicated that SCC for composite milk samples at 200 000 cells/mL was less able to detect IMI than at SCC for quarter milk samples at 200 000 cells/mL where the sensitivity was 70.8%. Although still less precise, results of the test at SCC of 150 000 cells/mL were more comparable with the results obtained in quarter milk samples (Tables 2 and 4). Specificity and the ability to detect culture-negative samples accurately using SCC were higher in composite samples than quarter milk samples. It increased from 66.8% (65.7%, 67.9%) at the SCC threshold of 150 000 cells/mL to 73.3% (72.2%, 74.5%) at 200 000 cells/mL compared to 63.6% (62.4%, 64.8%) for quarter milk sample at an SCC threshold of 200 000 cells/mL (Tables 2 and 4). Dohoo and Leslie (1991) reported in composite samples overall higher sensitivity (83.4%) and slightly lower specificity (58.9%) for major pathogens at 200 000 cells/mL. The SCC threshold point in composite samples where sensitivity and specificity were evenly balanced using the Youden’s index was indicated at 150 000 cells/mL (Table 2). This was found to be the optimum mathematical SCC threshold to identify IMI when examining composite samples without including any further information such as cow and environmental information.

The likelihood ratios were lower in composite samples than those at similar SCC thresholds in quarter milk samples. At 150 000 cells/mL and 200 000 cells/mL, the likelihood ratios to detect culture-negative composite samples were 1.034 and 0.864 compared to 1.177 in quarter samples at 200 000 cells/mL (Tables 2 and 4).

### Comparing quarter and composite cow milk sample results

The percentage of samples from which organisms were isolated at a threshold of 200 000 cells/mL was surprisingly similar in composite (18.31%) and quarter (18.73%) milk samples (Tables 1 and 3). It was, however, possible that the dilution effect of the composite milk sample caused more IMI to be at a subminimum level of detection for conventional laboratory microbiological detection systems (Dohoo & Leslie 1991; Pantoja et al. 2009; Schepers et al. 1997).

For every one infected composite or quarter milk sample at an SCC threshold of 50 000 cells/mL, 3.969 composite and 6.589 quarters were uninfected, respectively. At an SCC threshold of 200 000 cells/mL, the ratio of infected to uninfected was 1 to 2.268 in composite milk samples compared to 1 to 4.340 in quarter milk samples (Tables 1 and 3) making it more likely to correctly identify quarters with IMI using SCC.

Although specificity of the SCC test was higher in composite milk samples at a threshold of 200 000 cells/mL than for quarter milk samples, this difference between the sample types remained fairly constant at all SCC levels tested. Sensitivities of SCC for composite and quarter samples, however, started at a similar level at the 50 000 cells/mL SCC threshold (87.0% and 88.9%) but differed increasingly as the SCC level increased. Sensitivity decreased more rapidly in composite samples to 25.5% and quarter samples to 44.9% at the high SCC level (Tables 2 and 4).

An ‘optimum’ threshold was sought for the SCC test that could indicate IMI in milk samples at a level effective for screening of samples to be cultured. In composite milk samples, the best threshold level proved to be 150 000 cells/mL, whereas SCC threshold levels from 200 000 cells/mL were indicated for quarter milk (Tables 2 and 4). This result is in agreement with the 200 000 cells/mL threshold recommended by the National Mastitis Council when using the SCC test to indicate the presence of IMI in quarter milk samples (Smith et al. 2001). The AUC was found to be 0.7084 in composite and 0.7277 in quarter samples indicating that SCC was a good but not an excellent test for indicating IMI in both sample types (Table 5).

## Conclusion

The large data set used in this study was a reflection of actual samples received by the laboratory for analysis and interpretation. Samples were received from herds that differed in management levels, exposure to environmental conditions, microbial IMI and cow factors (age, parity, days in milk and milk yield).

The SCC test was found to be good but not excellent as an indicator of IMI in both quarter and composite sample types. The current threshold for SCC of 200 000 cells/mL used to detect only IMI in quarter milk samples was reconfirmed to be optimal. Based on the findings of this study, an SCC threshold of 150 000 cells/mL milk can be recommended for use in composite milk samples and could be used in practice as a selection criteria to select samples for culturing in large herds. This study indicated that the likelihood of identifying culture-negative samples at an SCC threshold of 200 000 cells/mL in quarter milk samples was 1.177 compared with 1.034 in composite milk samples at an SCC threshold of 150 000 cells/mL. Although less true culture-negative milk samples can be expected to be identified for composite milk compared to quarter milk samples because of a lower sensitivity of the SCC test in composite samples, it was indicated that SCC could be used at the lower threshold level to indicate IMI in composite milk samples.

It should be noted, however, that these optimal statistically determined SCC threshold levels need to be adapted depending on specific operational circumstances. Even at these ‘optimal’ SCC levels, using the SCC as the only test to predict the presence of IMI is helpful as screening test to select samples for microbiological determinations, but is not ideal as a stand-alone test to indicate IMI.
